# The Messy Alkaline Formose Reaction and Its Link to Metabolism

**DOI:** 10.3390/life10080125

**Published:** 2020-07-28

**Authors:** Arthur Omran, Cesar Menor-Salvan, Greg Springsteen, Matthew Pasek

**Affiliations:** 1 School of Geosciences, University of South Florida, Tampa, FL 33620, USA; mpasek@usf.edu; 2 Department Biologia, Universidad de Alcala, 28801 Madrid, Spain; cesar.menor@chemistry.gatech.edu; 3 Department of Chemistry, Furman University, Greenville, SC 29613, USA; greg.springsteen@furman.edu

**Keywords:** formose reaction, *Cannizzaro reaction*, formaldehyde, metabolism, metabolism first, proto-metabolism, chemical evolution, sugars, RNA world

## Abstract

Sugars are essential for the formation of genetic elements such as RNA and as an energy/food source. Thus, the formose reaction, which autocatalytically generates a multitude of sugars from formaldehyde, has been viewed as a potentially important prebiotic source of biomolecules at the origins of life. When analyzing our formose solutions we find that many of the chemical species are simple carboxylic acids, including α-hydroxy acids, associated with metabolism. In this work we posit that the study of the formose reaction, under alkaline conditions and moderate hydrothermal temperatures, should not be solely focused on sugars for genetic materials, but should focus on the origins of metabolism (via metabolic molecules) as well.

## 1. Introduction

### 1.1. A Brief History

Alexander Mikhaylovich Butlerov discovered formaldehyde (originally called dioxymethylene/methylene dioxide) in 1859 [1]. Dioxymethylene is the polymer of formaldehyde (paraformaldehyde). Butlerov isolated it from the formaldehyde polymer hexamethylene tetramine, by treatment with ammonia. Formaldehyde was conclusively identified with a molecular formula CH_2_O in 1869, by August Wilhelm von Hofmann [2,3].

Butlerov also discovered the formose reaction (also known as the Butlerov reaction) in 1861 [4]. In the original experiment he heated a solution of dioxymethylene mixed with quicklime (Ca(OH)_2_). The solution turned color and smelled sweet and of burnt sugar, so he tasted the solution and noticed it was sweet like sugar. Since its inception, the formose reaction has been synonymous with sugar production. Oskar Loew, who in the late 1880s improved the formose reaction, gave the “classic” form of the reaction and proposed that “formose” could be sorbose, an isomer of fructose (lävulose in the old German literature) [5]. Fischer and Baer discovered the polymerization of glyceraldehyde and/or dihydroxyacetone to yield sorbose and fructose, advancing our modern understanding during the early 1900s [6]. It is with a rich history in mind that many scientists see the formose reaction as the best candidate for the prebiotic synthesis of sugars.

### 1.2. A Plausible Place for the Formose Reaction

A plausible setting for the formose reaction may have existed in the Hadean epoch, 4.5–4.0 billion years ago (Ga) [7]. This was when the Earth accreted and cooled into a volcanic world with a thick, CO^2^ rich atmosphere and water ocean due to volcanic outgassing [7,8]. Geologic evidence from zircons has found that liquid water may have existed as long ago as 4.4 Ga, very soon after the formation of Earth [8].

Formaldehyde and glycolaldehyde might have been delivered by meteorites, especially during the heavy bombardment period. This hypothesis is supported by recent studies on the abundance of formaldehyde in interstellar space, comets, and interplanetary dust particles [9,10]. In addition, evidence exists for a photochemical and electrochemical production of formaldehyde in the CO_2_ dominated atmosphere of the early Earth [10,11,12]. Having available formaldehyde sources and ideal temperature ranges makes the early Earth—and other environments in our solar system—suitable for the formose reaction, given that the pH is agreeable locally [12].

### 1.3. The Modern Formose Reaction and Prebiotic Chemistry

In modern biology, sugars are found in genetic elements (nucleic acids) as part of the backbone, they provide structural support and energy storage polymers (polysaccharides), they promote cell- to-cell communication pathways (glycoproteins), and are a critical component of core metabolism (glycolysis, Calvin cycle, pentose phosphate pathway). This ubiquity in modern biology has led to a search for prebiotic sources of sugars available at the origins of life. In this context, the formose reaction gained prominence because at elevated temperatures and basic conditions, it autocatalytically generates a multitude of sugars from formaldehyde [12,13,14], including those with biological relevance. The formose reaction is classically catalyzed by calcium hydroxide and is catalyzed by other divalent metal ions as well [4,11,13,14,15,16]. The underlying mechanism involves aldol and retro-aldol reactions, and aldose–ketose isomerization with glycolaldehyde, glyceraldehyde, dihydroxyacetone, and tetrose sugars serving as important, early intermediates [4,11,13,14,15,16]. Also, it is important to note that a problem in the formose reaction is the rearrangements of sugars in alkaline solutions to form saccharinic acids, for example, glucose rearranges to hydroxymethy-l-3-deoxyribonic acid. In general, a complex variety of acids is obtained together with aldoses/ketoses [16,17]. Furthermore, sugar stability is often called into question raising concerns with formose chemistry [18]. Even though the formose reaction faces problems with rearrangements, and sugar stability, the formose reaction still may be the best candidate for the prebiotic synthesis of sugars.

Previous studies have shown that aspects of the formose reaction are geochemically plausible; however, concentrating mechanisms are required, and reactions from formaldehyde yield a complex mixture of sugars lacking appreciable amounts of any specific sugar, for example ribose [12,19,20].

While some formose reactions, such as a UV-catalyzed process can yield a primary product like pentaerythritol [19] our sugar yields lack specificity for a desired sugar species, such as ribose, which is typical of most formose reactions. This fits well into the current paradigm of the formose reaction, being nonspecific and requiring a high concentration of formaldehyde [12]. In general, low sugar yields (single digit percentages) are typical for the formose reaction [20,21,22].

**Figure 1:**
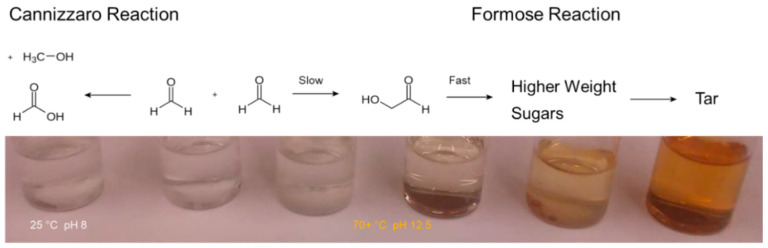
A classical view of the formose reaction. When heated under alkaline conditions formaldehyde solutions turn yellow in color. This yellowing is due to the production of sugars and their subsequent polymerization and dehydration into chromophoric species. The Cannizzaro reaction, which occurs concurrently, does not produce a color change.

When heated under alkaline conditions formaldehyde solutions turn yellow in color. This yellowing occurs due to the production of sugars from formaldehyde [4]. As the reaction proceeds the solution browns, which signals formaldehyde has been used up and some sugars have degraded into tar (Figure 1). The Cannizzaro reaction, a base-catalyzed disproportionation reaction, converts formaldehyde (C^0^) into formic acid (C^2+^) and methanol (C^2−^), also occurs concurrently [23,24,25,26,27,28,29].

The formose process is a set of two reactions. The first, a slow step, is the reaction of formaldehyde to form glycolaldehyde. The second reaction, which is fast and autocatalytic, involves the formation of many higher weight aldoses and ketoses from lower weight species. The primary interest in the formose reaction comes from the production of sugars and their possible link to an RNA world [7].

In this paper we wish to put forward the idea that the current perception of the function of the formose reaction in prebiotic chemistry may be incomplete. While sugars are produced, a main function of the formose reaction with regard to the origins of life may have been that it autocatalytically produced a set of proto-metabolites that were both simpler than typically depicted (a lower diversity of sugars) and more heterogeneous, also generating significant amounts of biologically relevant organic acids and hydroxy acids found at the core of modern metabolism. Thus, the formose reaction may have set the stage for a richer and more dynamic proto-metabolism than a mélange of sugars. The only organic component requirement for this system is formaldehyde, which acts as both the carbon source, and a redox agent [12].

## 2. Materials and Methods

### 2.1. Heated Alkaline Formaldehyde Solutions

Formose solutions were prepared using 100 mL of 0.32% (*w*/*v*) formaldehyde and 0.12% (*w*/*v*) methanol (Macron). Methanol was added as a stabilizer to prevent paraformaldehyde formation in solution. This dilution corresponds to a concentration of 1.0 M formaldehyde. Solutions contained either 0.5 g CaCO_3_ (Sigma Aldrich, St. Louis, MI, USA) or were run without CaCO_3_. First, the solutions were titrated with sodium hydroxide (Sigma Aldrich) to the desired pH of 12.5 before heating. Second, for hydrothermal synthesis of the formose sugars, the flask tops were covered with aluminum foil and autoclaved for 1 h at 120 °C and 200 kPa.

Control experiments were performed to ensure we indeed were running the formose reaction and that sugars under these conditions were forming our metabolic components (see Supplementary Materials). Glucose solutions at pH 12.5 were heated at 120 °C to see if the same products formed as detected in our formose reactions (Figure S1).

Some experiments required observation and sampling of the formose solutions in real time in order to validate we were running the autocatalytic formose reaction (Figure S2). These experiments were run at lower temperature heating conditions (80 °C) that are observable with the naked eye. These reactions were observed for yellowing (as an indication of the reaction producing sugars); the production of formose products was verified spectroscopically with NMR at different time points during heating (Figure S2a–c). Formaldehyde solutions were loaded into a hot water bath placed on a hotplate (Corning 6795 Stirring Hot Plate) in the fume hood. The temperature of the water bath was monitored and controlled with an attached thermocouple sensor.

### 2.2. Chemical Characterization of Formose Products

Filtered product solutions were characterized using 1D proton magnetic resonance measurements with ZGGPW5 or ZGESGP water suppression pulses on a Bruker 600 MHz NMR or Bruker Neo 600 MHz NMR. 100 mM 4,4-dimethyl-4-silapentane-1-sulfonic acid (DSS, Sigma-Aldrich) and 10% by volume D_2_O were used to standardize (at 0 ppm) a reference point and lock spectral measurements. Scans were run for about 10 min at 100 normal scans.

In addition, glycosyl composition analysis was performed by combined gas chromatography/mass spectrometry (GC/MS) of the per-O-trimethylsilyl (TMS) derivatives of the monosaccharide methyl glycosides produced from the sample by acidic methanolysis. This method of acidic methanolysis reacts all sugars to their aldol forms and then detects them via a GC/MS run against sugar standards, and an extensive sugar library. The dried formose sample (1.8 mg) with inositol (internal standard, 20 μg) was heated with methanolic HCl in a sealed screw-top glass test tube for 18 h at 80 °C. After cooling and removal of the solvent under a stream of nitrogen, the samples were treated with a mixture of methanol, pyridine, and acetic anhydride for 30 min. The solvents were evaporated and the samples derivatized with Tri-Sil (Pierce) at 80 °C for 30 min. GC/MS analysis of the TMS methyl glycosides was performed on an Agilent 7890A GC interfaced to a 5975C MSD, used a Supelco Equity-1 fused silica capillary column (30 m × 0.25 mm ID). The mixture of derivatized sample was analyzed by GC/MS and per-O-trimethylsilyl derivatives of monosaccharide residues run alongside the sample and used for quantification of each monosaccharide residue. The peak intensity of GC/MS was used for quantification compared to each standard after calculating the response factor for each residue.

## 3. Results and Discussion

**Figure 2:**
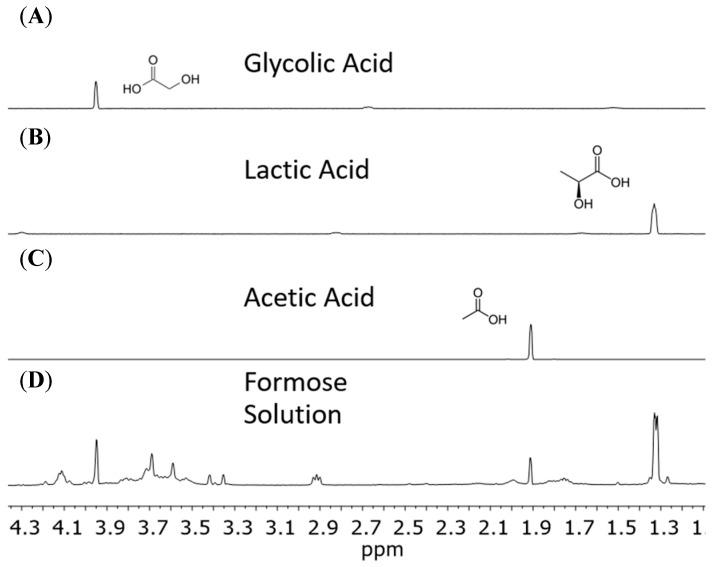
Standards of carboxylic acids compared to a formose solution using proton NMR. Standards are at 10 mM in a pH 7.5 solution with DSS and D_2_O. Spectra (**A**–**C**) represent standard glycolic acid, lactic acid, and acetic acid, respectively. The magnified spectrum (**D**) represents a formose reaction run at pH 12.5 and 120 °C catalyzed using Ca^2+^. Formose solution was titrated with HCl down to a pH of 7.5 after autoclaving and cooling down to room temperature.

**Figure 3:**
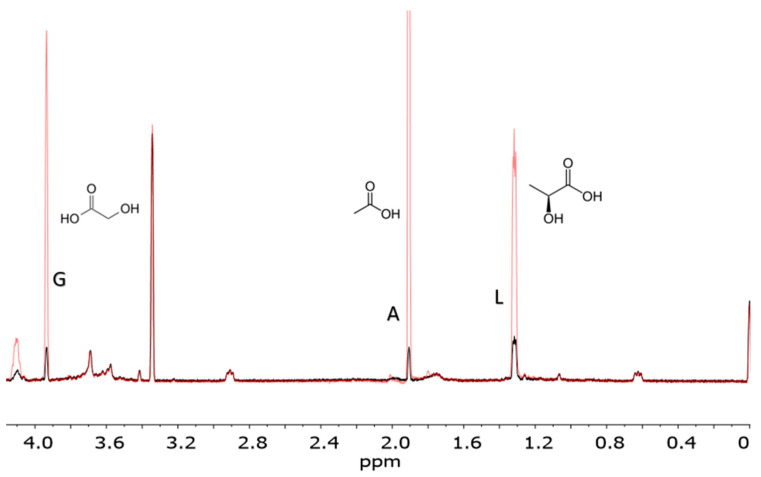
Carboxylic acids spiked into a formose solution. The red spectral lines represent the spiked-in set, and include added lactic acid, glycolic acid, and acetic acid to a formose solution. The black spectral lines represent the formose products with no additional carboxylic acids spiked-in. Spectra were measured from a formose reaction run at pH 12.5 and 120 °C catalyzed using Ca^2+^.The abbreviations G, A, and L stand for glycolic acid, acetic acid, and lactic acid, respectively. This reaction was performed at pH 12.5, 120 °C, the product solution was cooled and titrated to pH 7.5 before NMR analysis.

When analyzing our formose yields, from pH 12.5 and temperatures 80 °C and 120 °C, we found the main type of chemical species were carboxylic acids, including α-hydroxy acids, based on our NMR results. Reactions heated to 80 °C and 120 °C contained the same products resulting in very similar spectra. We saw formic acid, acetic acid, glycolic acid, and lactic acid in solution (Figure 2 and Figure 3). Figure 2 and Figure 3 indicate the identification and verification of lactic acid, acetic acid, and glycolic acid via spiked-in NMR experiments.

While the sugars were produced directly by the formose reaction, formic acid is a product of the Cannizzaro reaction between two equivalents of formaldehyde. The faint signatures for sugars are the magnified peaks (insets of Figure S2b,c) showing the convoluted “sugar peak region” from 3.2 to 4.4 ppm (Figure S2a–c). The Cannizzaro products were observed in solution using NMR, we see formic acid (8.4 ppm) and methanol (3.2 ppm) (Figure S2a). Another indicator of the Cannizzaro process is the decrease in pH over time, from 12.5 initially to ~8 by the end of the reactions. As our reactions start with methanol as an added stabilizer we rely on the production of formic acid to verify the Cannizzaro process is occurring in solution. Methanol is generated also by this process, and by the reaction of formaldehyde and C2/C3 sugars (also a Cannizzaro) to generate the carboxylic acid products. In basic solution formaldehyde could undergo the Cannizzaro reaction generating additional methanol and formic acid regardless of heating or other reactions occurring. It seems at elevated temperature and pH (80+ °C and 12+ respectively) most sugars further react to give the more stable Cannizzaro products, which we inspect at equilibrium. Only ~8 × 10^−5^% moles formaldehyde remained as sugars upon analysis.

The ratio of the non-sugar to sugar species in our solutions was about 99+% to 1−%, respectively. The mol% values were calculated by integrating our peaks from the NMR experiments and back calculating using our initial concentration of formaldehyde (100 mmol) (Figure 2 and Figure 3). In general, we saw 63.1 mol% formaldehyde converted to organic acids in total at equilibrium. This was primarily lactic acid at 41.0 mol%, with 7.4 mol% formic acid, 7.4% glycolic acid, and 7.3 mol% acetic acid as well. We also observed a complex mixture of low mass alcohols (1–3 carbons long) in solution, at 35.6 mol% (excluding initial methanol concentration). These include ethylene glycol, and glycerol. If concentrating sour solutions before analysis the methanol may evaporate, resulting in a fluctuation in peak intensity. Lastly, we observed 1.1 mol% methoxy methanol, formed from Cannizzaro products further reacting in solution, and not due to the formose process.

Realistically, the formose process in nature would have been very messy, especially in alkaline waters. Alkaline water is the main form of water that results from water-rock interactions (e.g., serpentinization), and this basic solution is required for the formose and Cannizzaro reactions. The reaction would have had other carbon species as contaminants. Also, other elements might have been incorporated into the process, other than just C, H, and O. Additionally, the formose process would come into contact with various metals/salts in nature. In order to see what the formose sugar yields looked like uncatalyzed (only in the presence of NaOH), or in the absence of divalent metal cations, we performed hydrothermal alkaline formose reactions at 80 °C with no calcium salts added.

**Figure 4:**
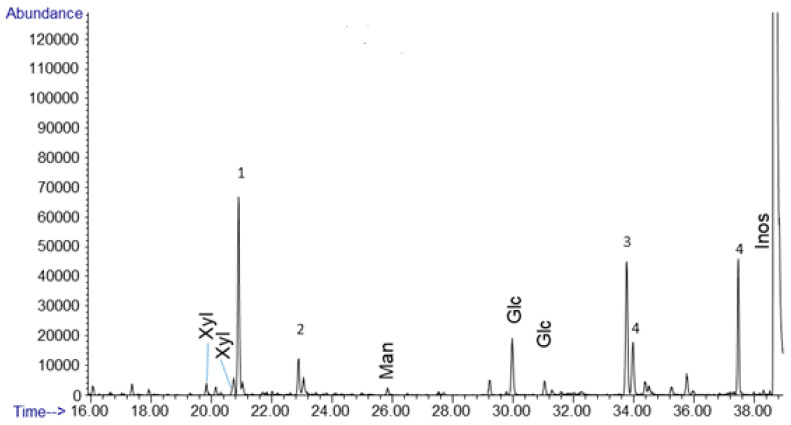
Gas chromatogram for an uncatalyzed formose reaction. 1 M formaldehyde w/0.5 M methanol at pH 12.5 heated at 80 °C for 1 h. Glc, Man, and Xyl, represent glucose, mannose, and xylose, respectively. Peaks labeled 1 and 2 are organic acids coeluting with xylose, likely including saccharinic acids. Peak 3 is sorbitol and peak 4 is myo-inositol, these were formed from the inositol standard, not the formose reaction.

Of the sugars produced by an alkaline formaldehyde solution containing no calcium or any other divalent metal cation, we see the primary sugar produced is glucose at 79.3 mol% of the sugar yield (Figure 4, Table 1). For the sample analyzed (1.8 mg crude dried mass) sugar yield was 0.003% of the total mass, a fairly low % yield for the reaction with respect to hexoses. Glucose is important because it functions as the core sugar in modern energy metabolism. Other detectable products included mannose, and xylose, at 6.25 and 14.5 mol% of the sugar yield, respectively (Figure 4, Table 1). Calculated mol% for sugars were based on the detected glucose, xylose, and mannose counted in the GC/MS quantification. Our results, analyzed via derivatization and GC/MS, relied on acid hydrolysis, which cause ketoses to become indistinguishable from aldoses in the dried mass used for GC/MS. We also ran controls on calcium catalyzed formose reactions and noted the sugar yields were an order of magnitude higher. This still was less than 1% of the dried crude mass. The calcium-catalyzed and no calcium formose reactions also exhibited the same organic acids and alcohols; we did not observe any difference in these products.

**Figure 5:**
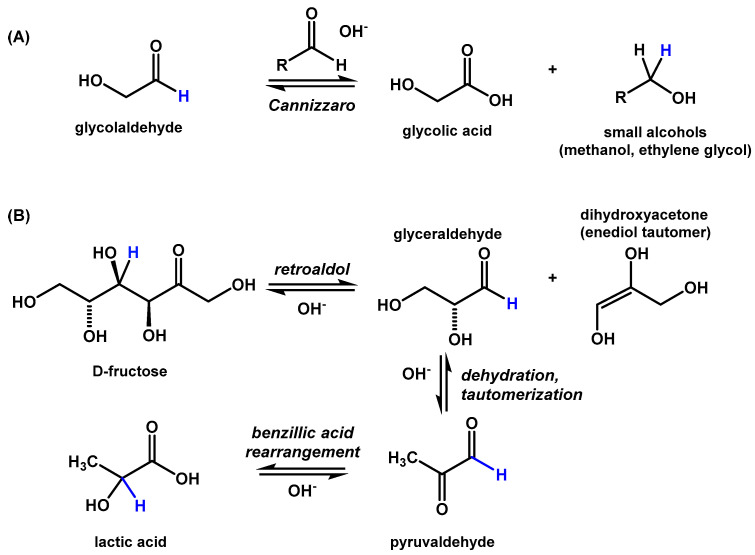
Reaction scheme for the formation of glycolic and lactic acids. (**A**) Glycolaldehyde is transformed into glycolic acid by a base-catalyzed Cannizzaro reaction with an additional small aldehyde, for example, formaldehyde or a second equivalent of glycolaldehyde. (**B**) Fructose is transformed into lactic acid by a retroaldol reaction followed by a benzillic acid rearrangement.

Within studies of biomass conversion, the formation of carboxylic acids, including lactic acid, glycolic acid, and acetic acid, has been observed in high-temperature alkaline reactions starting from glucose [30], fructose [31,32], pyruvaldehyde [32,33], and glycolaldehyde [34]. Glycolic acid is expected to be generated through a Cannizzaro reaction of glycolaldehyde with formaldehyde (or other small aldehydes, Figure 5A). Lactic acid is likely generated as a result of a benzillic rearrangement of pyruvaldehyde that was generated through the dehydration and tautomerization of glyceraldehyde (Figure 5B). Glyceraldehyde is produced both by the reaction of glycolaldehyde with formaldehyde in the formose reaction, and the retro-aldol breakdown of higher sugars such as fructose.

Additionally, we ran controls on glucose degradation at 120 °C and pH 12.5 to check retro-aldol cleavage of sugars to explain the formation of lactic acid, and Lobry de Bruyn-van Ekenstein rearrangements [32,33]. The final controls were performed to make sure O_2_ was not interfering with our reaction [35]. Both oxygenated and anoxic experiments show the formation of organic acids, polyols, and sugars; albeit, oxygenated experiments have longer induction periods and slower sugar production [35]. These control groups support our hypothesis, showing that sugar degradation and Lobry de Bruyn-van Ekenstein rearrangements were responsible for our products formed and that O_2_ in the atmosphere was not heavily interfering, allowing for the Cannizzaro process to produce additional products.

## 4. Conclusions

The formose reaction is often depicted as a source of biologically relevant sugars, such as ribose, along with a messy assortment of other sugars. It is affected by many factors in its environment, such as temperature, alkalinity, minerals, and clays [36,37,38,39]. The process of chemical selection (natural selection acting on populations of chemical species) could “find” a good fit for a sugar, such as ribose, over time using factors in the environment such as minerals, borates, calcium, reactive phosphates, etc. [15,37,38,39,40]. There are product stability issues for the formose system, or for sugars in general, based on higher heat and pH conditions [18]; however, it is under these conditions that we see products useful for metabolism, and thus for life. This result may bring a new perspective and highlights a different but important feature of the formose reaction and actually shows it to be a diverse and flexible promoter of biomolecule synthesis.

While not actually chaotic, the formose reaction is indeed a complex system, as far as chemical systems are concerned. It seems the proper view of the formose system, coupled to the Cannizzaro system, is that they produce a complex mixture of organic acids, polyols, and sugars together [35]. The chemistry is unruly and unspecific for the formose reaction, moreover it is inextricably linked to the Cannizzaro reaction further complicating matters. When this complex system of reactions is given a very alkaline environment and moderate hydrothermal temperatures it yields a plethora of metabolically relevant products (Figure 2, Figure 3 and Figure 4).

Our results show that many simple carboxylic acids, including α-hydroxy acids, associated with metabolism are produced by the formose process (Figure 2 and Figure 3). The concurrent Cannizzaro process, a more orderly system, gives additional metabolic components, specifically and reliably, namely the compounds formic acid and methanol [27,28,29]. The Cannizzaro system may also be the thermodynamically preferred trap for the products of the messy and convoluted systems. Finally, the sugars that are produced from a formose process that lacks a catalyst (or is catalyzed by NaOH) yields almost 80 mol% glucose (Figure 4, Table 1). Glucose is fundamental to core metabolism and energy flow within life. Also, other non-enzymatic metabolic studies require a glucose source for non- enzyme-based glycolysis and pentose-5-phosphate pathways [41,42], which the formose reaction may be able to provide as a proto-metabolic system. Moreover, less than 1% of our starting formaldehyde remained as sugars in solution after undergoing heating and high pH conditions; the thermodynamic minimum for the formose process under these conditions would be in the form of carboxylic acids rather than sugars, especially if oxygen is present, but also in anoxic conditions as well.

It is with all this considered that the “purpose” that comes to mind when contemplating the formose process should not be exclusively sugars-to-ribose, ribose-to-RNA first but could also be, metabolic components setting up protometabolic systems, which given the time and energy necessary for chemical evolution, would one day form a genetic material such as RNA. We believe this to be in support of an RNA world hypothesis, which is a sturdy and well-buttressed hypothesis standing the test of time [43]. Having said that it is very interesting that, currently, the only known abiotic autocatalytic cycle is the formose reaction [44]. This fact may be more important in understanding the formose reaction in terms of prebiotic significance and chemical evolution, than the fact that current genetic materials have a ribose-based structure. Under alkaline conditions and moderate hydrothermal temperatures, the formose reaction may have been the first step to life from an emergence of metabolism perspective.

## Figures and Tables

**Table 1:** Uncatalyzed formose reaction sugars. 1 M formaldehyde w/0.5 M methanol at pH 12.5 heated at 80 °C for 1 h. Sugars produced and clearly identifiable are listed and quantified by mass and mol% (with respect to total sugars generated). Sugars include glucose at nearly 80% of the produced sugar yield, with small amounts of xylose and mannose present as well. The sample analyzed was 1.8 mg of dried crude reaction mixture, sugar yield was 0.003%.

Sugar	Molecular Weight (g/mol)	Weight (µg)	Mole %	Molecular Formula
Glucose	180.2	0.044	79.3	C_6_H_12_O_6_
Xylose	150.1	0.007	14.5	C_5_H_10_O_5_
Mannose	180.2	0.003	6.2	C_6_H_12_O_6_

## Supplementary Materials

The following are available online at https://www.mdpi.com/2075-1729/10/8/125/s1, Figure S1: NMR verification of the breakdown of glucose and formation of lactic acid. Figure S2: Time point verifications for formose reactions.
