# Supplementary material for: The Messy Alkaline Formose Reaction and Its Link to Metabolism
Source: Life (Basel). 2020 Jul 28;10(8):125. doi: 10.3390/life10080125 (PMC7460143; doi:10.3390/life10080125)
Supplement: Supplementary file 1 [file life-10-00125-s001.pdf]

Supplemental Information

By Arthur Omran

Additional Methods:

Controls were run to identify formose reaction products in the absence of oxygen. First, 10 mL solution of 0.32% (w/v) formaldehyde and 0.12% (w/v) methanol (Macron) was prepared. This dilution corresponds to a concentration of 1.0 M formaldehyde and was sealed in borosilicate vials and capped. The solution was titrated with sodium hydroxide to the desired pH of 12.5 before heating and then bubbled under Argon gas (Airgas) for 10 minutes. Secondly, for hydrothermal synthesis of the formose sugars, formaldehyde solutions were heated at 120 °C for 30 minutes in a bench top oven (Quincy Labs).

Additional controls were run to identify if simple carboxylic acids form from the reactions of sugars in the hot alkaline solutions in the absence of oxygen and formaldehyde. First, 10 mL of 1.5 M glucose solution was prepared. The solution was titrated with sodium hydroxide to the desired pH of 12.5. The solution was bubbled under Argon gas (Airgas) for 10 minutes and was sealed in borosilicate vials and capped. Secondly, for hydrothermal synthesis of the carboxylic acids, glucose solutions were heated at 120 °C for 30 minutes in a bench top oven (Quincy Labs).

Additional Results:

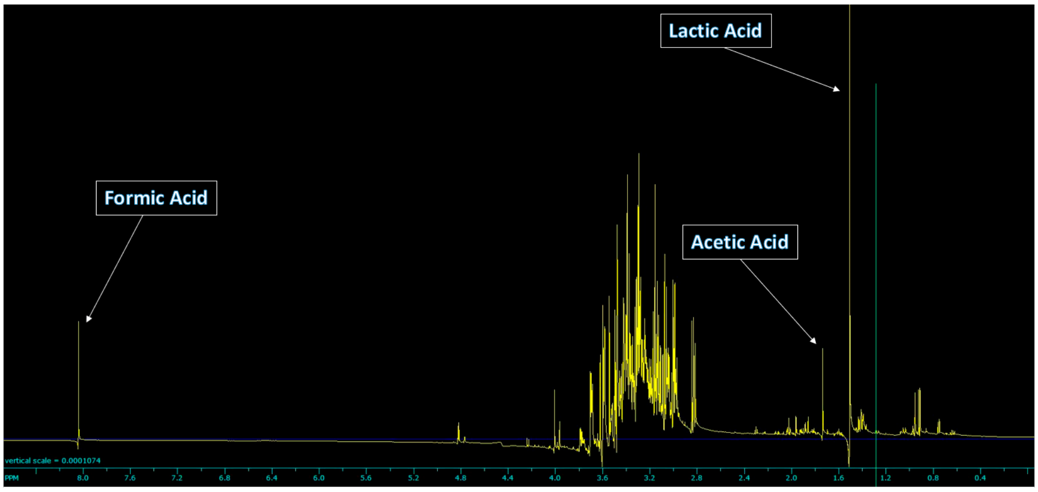

Figure S1. In this experiment we see the breakdown of glucose and formation of lactic acid, etc. This control was run in the absence of oxygen and without any formaldehyde. Slight peak shifts are due to a difference in pH, samples were not titrated to pH 7.5 before NMR.

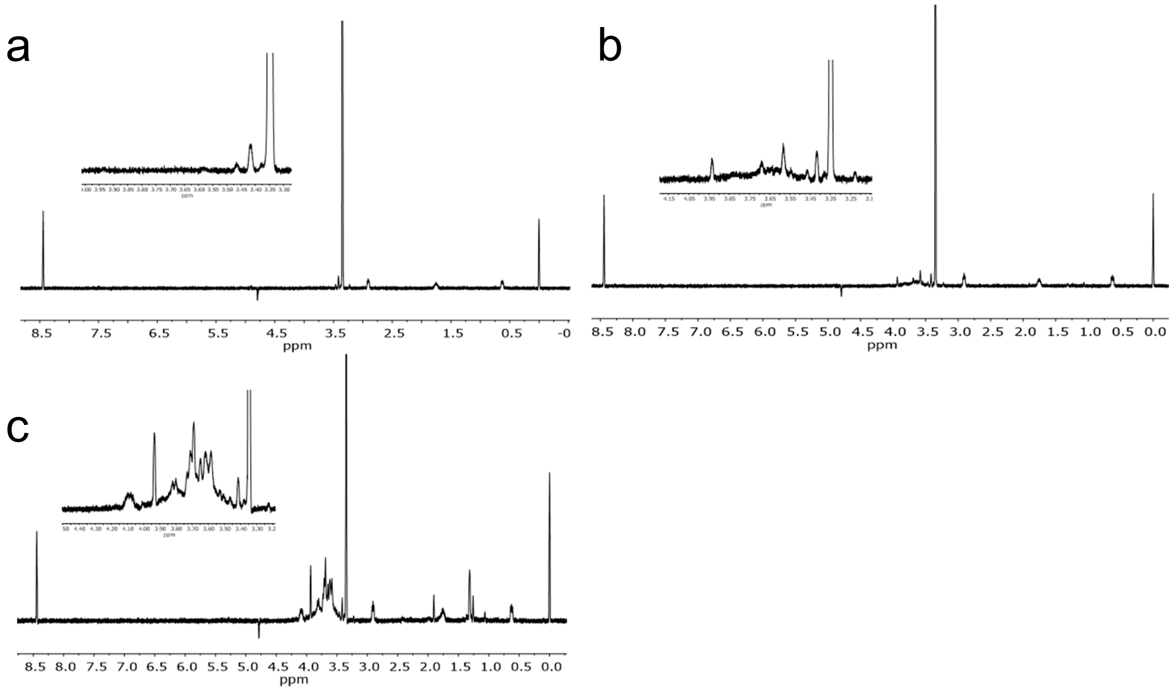

Figure S2. Time Points. 80°C. (a) early time point at 10 minutes, solution clear only products are Cannizzaro products (formic acid, methanol and methoxymethanol). A true negative result for the formose reaction. (b) Middle time point, at 15 minutes, solution clear, formose products and Cannizzaro products in solution. Can be interpreted as a false negative if NMR was not performed. (c) Late Time point, 30 minutes, more formose products in solution. This serves as a true positive result. The insets of a-c are of range 3.5-.4.0 ppm to look for sugar signatures.
